# Prognostic significance of combined pretreatment lymphocyte counts and body mass index in patients with head and neck cancer treated with radiation therapy

**DOI:** 10.1002/cam4.1489

**Published:** 2018-05-23

**Authors:** Yao‐Yu Wu, Kai‐Ping Chang, Chien‐Yu Lin, Ping‐Ching Pai, Hung‐Ming Wang, Cheng‐Lung Hsu, Chun‐Ta Liao, Tzu‐Chen Yen, Tuan‐Jen Fang, Shiang‐Fu Huang, Chung‐Jan Kang, Ku‐Hao Fang, Wan‐Ni Lin, Yu‐Chien Wang, Li‐Jen Hsin, Ngan‐Ming Tsang

**Affiliations:** ^1^ Department of Radiation Oncology Linkou Chang Gung Memorial Hospital and Chang Gung University Taoyuan City Taiwan; ^2^ Department of Otorhinolaryngology, Head and Neck Surgery Linkou Chang Gung Memorial Hospital and Chang Gung University Taoyuan City Taiwan; ^3^ Division of Hematology‐Oncology Department of Internal Medicine Linkou Chang Gung Memorial Hospital and Chang Gung University Taoyuan City Taiwan; ^4^ Department of Nuclear Medicine and Molecular Imaging Center Linkou Chang Gung Memorial Hospital and Chang Gung University Taoyuan City Taiwan; ^5^ School of Traditional Chinese Medicine Chang Gung University Taoyuan City Taiwan

**Keywords:** Body mass index, head and neck cancer, lymphocyte count, prognosis, radiotherapy

## Abstract

We aimed to investigate the prognostic significance of combined pretreatment lymphocyte counts (LCs) and body mass index (BMI) in patients with head and neck cancer (HNC) treated with radiation therapy (RT). Nine hundred and twelve patients with HNC who were treated with RT were retrospectively reviewed. Survival was analyzed by stratifying the patients according to pretreatment LCs and BMI. Patients with low pretreatment LCs and BMI were characterized by a more advanced T stage, fewer nasopharyngeal subsites, less smoking and drinking, and fewer comorbidities. Patients with low pretreatment LCs and BMI had a significantly poorer overall and distant metastasis‐free survival than those with high pretreatment LCs and BMI. No significant differences were observed in terms of local or regional recurrence‐free survival. Combined pretreatment LCs and BMI may be more effective at predicting overall and distant metastasis‐free survival in patients with HNC treated with RT.

## Introduction

Head and neck cancer (HNC) is an aggressive disease that represents the sixth most common cancer worldwide, with approximately 640,000 new patients diagnosed annually, resulting in nearly 390,000 deaths each year 1. Although the tumor‐node‐metastasis staging system has been used for prognostic prediction for >30 years, in many instances, the outcomes were still unpredictable. Consequently, there is a need to explore other potential prognostic factors.

The reported prevalence of malnutrition in patients with HNC is approximately 50.0% 2, with low lymphocyte counts (LCs) and body mass index (BMI) considered as predictors of a poor nutritional status 3, 4. Several studies have investigated an association between pretreatment BMI and survival outcomes in patients with HNC 5, 6 and reported that a high BMI is associated with a favorable survival outcome.

Lymphocytes constitute approximately one‐third of all human white blood cells. They play a critical role in the immune response to cancer 7. Studies 8, 9, 10, 11, 12, 13 have shown that lymphopenia can predict survival in several types of cancers, including HNC. Despite many studies 5, 6, 8, 9, 10, 11, 12, 13 having identified lymphopenia or BMI as prognostic factors, no studies have investigated an association between pretreatment LCs and BMI in patients with HNC treated with definitive radiation therapy (RT). Therefore, the purpose of this study was to investigate the prognostic significance of combined pretreatment LCs and BMI in patients with HNC treated curatively with RT alone or concurrent chemoradiotherapy (CCRT).

## Materials and Methods

### Patients

The medical records of patients with HNC (nasopharyngeal, oropharyngeal, laryngeal, or hypopharyngeal cancer; *n *=* *912), who were treated with RT alone or CCRT at the Linkou Chang Gung Memorial Hospital (Taoyuan City, Taiwan) between January 2005 and December 2012, were retrospectively reviewed. All patients had histologically proven HNC and had undergone complete staging according to the 2010 American Joint Committee on Cancer Tumor‐Node‐Metastasis Staging System. Exclusion criteria included the following: (1) a second primary cancer occurring within 3 years prior to or after primary cancer treatment, (2) an equivalent dose in 2 Gy fractions (EQD2) of <60.0 Gy, (3) the unavailability of official pathological reports, (4) an age <18 years, and (5) a treatment duration of ≥70 days. Data were collected by a radiation oncologist and an experienced nurse. The study protocol was approved by the Institutional Review Board Committee of the Linkou Chang Gung Memorial Hospital, Taoyuan City, Taiwan (approval no.: IRB201700249B0). The need for informed consent was waived due to the retrospective nature of the study. Research was conducted in accordance with Helsinki Declaration of 1975, as revised in 1983.

### Measurements of pretreatment lymphocyte counts and body mass index

Pretreatment hematological testing and body weight measurements were conducted within 14 days prior to the commencement of RT. Pretreatment LCs were measured by multiplying the white blood cell count by the percentage of lymphocytes in a complete blood cell count test. The pretreatment BMI was calculated as the pretreatment body weight in kilograms divided by the square of the height in meters.

### Variable definitions

The treatment duration was defined as the time interval between the first and last RT session. Performance status was determined using the Eastern Cooperative Oncology Group scale. The presence of comorbidities (dichotomized as “yes” or “no”) was assessed using the Charlson Comorbidity Index 14. Cigarette smoking was dichotomized as “yes” (subjects who smoked ≥ 100 cigarettes in their lifetime) or “no” (subjects who smoked < 100 cigarettes in their lifetime and are currently not smoking) according to the American Centers for Disease Control and Prevention classification system 15. Similarly, alcohol consumption (current or former drinkers vs. nondrinkers) and betel quid chewing (current or former chewers vs. nonchewers) were treated as dichotomized variables.

### Statistical analyses

The primary outcome of this study was overall survival (OS). Secondary outcomes included local recurrence‐free survival (LRFS), regional recurrence‐free survival (RRFS), and distant metastasis‐free survival (DMFS). Patients were stratified according to pretreatment LCs and BMI: Group 1 (low pretreatment LCs and a low pretreatment BMI), Group 2 (high pretreatment LCs and a low pretreatment BMI), Group 3 (low pretreatment LCs and a high pretreatment BMI), and Group 4 (high pretreatment LCs and a high pretreatment BMI). The median LC of this study cohort and a BMI of 25.0 kg/m^2^ were used as the cutoff values. OS was calculated as the time elapsed (in months) from the date of commencing RT to the date of death. Differences between the groups were assessed by Student’s *t* tests (continuous variables) or chi‐square tests (categorical variables). Survival curves were plotted using the Kaplan–Meier method and compared using the log‐rank test. Multivariate Cox proportional hazards regression analyses were used to identify independent predictors of OS. The results are expressed as the hazard ratios (HRs) with 95.0% confidence intervals. The variables included in the risk factor analysis were age, sex, the American Joint Committee on Cancer stage, primary tumor site, pretreatment LCs and BMI, cigarette smoking, betel quid chewing, alcohol consumption, the presence of comorbidities, CCRT, the use of positron emission tomography (PET) for staging, and the EQD2. Two‐tailed *P* values <0.05 were considered statistically significant.

## Results

### Patient characteristics

The demographic and clinicopathological characteristics of the patients are summarized in Table 1. The median follow‐up duration of the survivors was 5.5 years. The median age of the patients was 49.8 (range, 18.4–86.7) years. The median duration of RT treatment was 53 (range, 44–70) days. Seven hundred and twenty‐seven patients (79.7%) underwent pretreatment PET for staging, and 780 patients (85.5%) were treated with CCRT.

**Table 1:** Patient characteristics

BMI/LC	Group 1 (*n *=* *315)	Group 2 (*n *=* *255)	Group 3 (*n *=* *141)	Group 4 (*n *=* *201)	All (*n *=* *912)	*P*‐value
LC, cells/μL						
Median (range)	1481 (345–1944)	2366 (1956–5445)	1588 (342–1949)	2413 (1952–5175)	1950 (342–5445)	<0.001^a^
Mean (±SD)	1445 ± 343	2512 ± 554	1515 ± 322	2549 ± 554	1997 ± 702	
BMI, kg/m^2^						
Median (range)	21.7 (12.9–25.0)	22.3 (14.9–25.0)	27.2 (25.0–48.8)	27.1 (25.0–43.8)	23.7 (12.9–48.8)	<0.001^a^
Mean (±SD)	21.4 ± 2.2	21.9 ± 2.2	27.6 ± 2.7	27.8 ± 2.6	23.9 ± 3.8	
Age, years						
Median (range)	49.6 (18.4–86.7)	49.2 (19.3–81.3)	50.6 (18.9–79.7)	50.5 (28.5–80.7)	49.8 (18.4–86.7)	0.499^a^
Mean (±SD)	50.6 ± 12.5	49.9 ± 10.8	51.7 ± 10.6	50.8 ± 10.8	50.6 ± 11.4	
Treatment duration, days						
Median (range)	53 (45–70)	53 (44–69)	53 (45–69)	52 (44–67)	53 (44–70)	0.709^a^
Mean (±SD)	53.7 ± 4.1	53.9 ± 4.3	53.5 ± 4.2	53.5 ± 4.4	53.7 ± 4.2	
EQD2, Gy						
Median (range)	72 (66–76)	72 (66–76)	72 (68–76)	72 (66–76)	72 (66–76)	0.357^a^
Mean (±SD)	71.7 ± 2.0	71.6 ± 1.8	71.9 ± 1.7	71.5 ± 1.7	71.7 ± 1.8	
2010 AJCC T stage, *n* (%)						
T1–2	122 (38.7%)	110 (43.1%)	66 (46.8%)	108 (53.7%)	406 (44.5%)	0.009^a^
T3–4	193 (61.3%)	145 (56.9%)	75 (53.2%)	93 (46.3%)	506 (55.5%)	
2010 AJCC N stage, *n* (%)						
N0–1	132 (41.9%)	114 (44.7%)	69 (48.9%)	100 (49.8%)	415 (45.5%)	0.279^b^
N2–3	183 (58.1%)	141 (55.3%)	72 (51.1%)	101 (50.2%)	497 (54.5%)	
Primary tumor site, *n* (%)						
Nasopharynx	160 (50.8%)	119 (46.7%)	85 (60.3%)	132 (65.7%)	496 (54.4%)	0.004^b^
Oropharynx	69 (21.9%)	60 (23.5%)	29 (20.6%)	30 (14.9%)	188 (20.6%)	
Hypopharynx	66 (21.0%)	52 (20.4%)	19 (13.5%)	24 (11.9%)	161 (17.7%)	
Larynx	20 (6.3%)	24 (9.4%)	8 (5.7%)	15 (7.5%)	67 (7.3%)	
Sex, *n* (%)						
Female	65 (20.6%)	34 (13.3%)	23 (16.3%)	32 (15.9%)	154 (16.9%)	0.132^b^
Male	250 (79.4%)	221 (86.7%)	118 (83.7%)	169 (84.1%)	758 (83.1%)	
BMI, kg/m^2^, *n* (%)						
<25.0	315 (100.0%)	255 (100.0%)	0 (0.0%)	0 (0.0%)	570 (62.5%)	<0.001^b^
≥25.0	0 (0.0%)	0 (0.0%)	141 (100.0%)	201 (100.0%)	342 (37.5%)	
Cigarette smoking, *n* (%)						
No	119 (37.8%)	51 (20.0%)	62 (44.0%)	60 (29.9%)	292 (32.0%)	<0.001^b^
Yes	196 (62.2%)	204 (80.0%)	79 (56.0%)	141 (70.1%)	620 (68.0%)	
Betel quid chewing, *n* (%)						
No	202 (64.1%)	143 (56.1%)	89 (63.1%)	120 (59.7%)	554 (60.7%)	0.234^b^
Yes	113 (35.9%)	112 (43.9%)	52 (36.9%)	81 (40.3%)	358 (39.3%)	
Alcohol consumption, *n* (%)						
No	180 (57.1%)	112 (43.9%)	77 (54.6%)	108 (53.7%)	477 (52.3%)	0.014^b^
Yes	135 (42.9%)	143 (56.1%)	64 (45.4%)	93 (46.3%)	435 (47.7%)	
Comorbidities, *n* (%)						
No	213 (67.6%)	162 (63.5%)	73 (51.8%)	100 (49.8%)	548 (60.1%)	<0.001^b^
Yes	102 (32.4%)	93 (36.5%)	68 (48.2%)	101 (50.2%)	364 (39.9%)	
Chemotherapy, *n* (%)						
No	48 (15.2%)	42 (16.5%)	16 (11.3%)	26 (12.9%)	132 (14.5%)	0.481^b^
Yes	267 (84.8%)	213 (83.5%)	125 (88.7%)	175 (87.1%)	780 (85.5%)	
PET, *n* (%)						
No	69 (21.9%)	48 (18.8%)	25 (17.7%)	43 (21.4%)	185 (20.3%)	0.666^b^
Yes	246 (78.1%)	207 (81.2%)	116 (82.3%)	158 (78.6%)	727 (79.7%)	

AJCC, American Joint Committee on Cancer; BMI, body mass index; EQD2, equivalent dose in 2 Gy fractions; LC, lymphocyte count; PET, positron emission tomography; SD, standard deviation.

a ANOVA.

b Chi‐square test.

The median pretreatment LC for all patients was 1950 (range, 342–5445) cells/μL. The median pretreatment BMI was 23.7 (range, 12.9–48.8) kg/m^2^. In total, 342 patients (37.5%) had a pretreatment BMI of ≥25.0 kg/m^2^. Group 1 patients with low pretreatment LCs and a low pretreatment BMI were characterized by a more advanced T stage (*P *<* *0.01), fewer nasopharyngeal subsites (*P *<* *0.01), less smoking (*P *<* *0.001) and drinking (*P *<* *0.05), and fewer comorbidities (*P *<* *0.001). No significant differences were observed with respect to age, sex, N stage, betel quid chewing, CCRT, treatment duration, the use of PET for staging, or the EQD2.

### Overall survival outcomes

**Figure 1:**
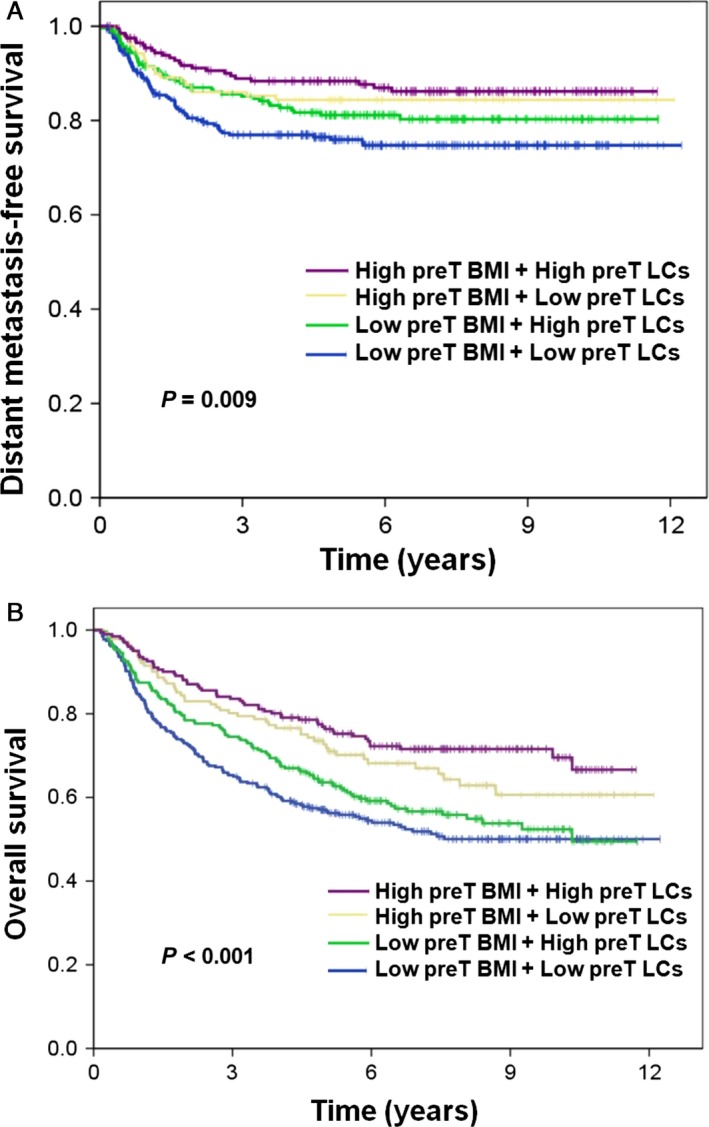
Kaplan–Meier curves of (A) distant metastasis‐free survival and (B) overall survival in patients with head and neck cancer stratified according to pretreatment (preT) lymphocyte counts (LCs) and body mass index (BMI).

The 5‐year OS rate of all patients was 65.5%. The 5‐year OS rates for Group 1, Group 2, Group 3, and Group 4 patients were 56.8%, 63.3%, 72.3%, and 76.3%, respectively (*P *<* *0.001; Fig. 1). In the univariate analysis, pretreatment LCs and a pretreatment BMI, either alone or in combination, were significant predictors of OS (Fig. 1 and Table 2). Other factors, including age, sex, T/N stage, primary tumor site, cigarette smoking, betel quid chewing, alcohol consumption, the presence of comorbidities, CCRT, the use of PET for staging, and the EQD2, were also significant predictors of OS (Table 2). In the multivariate analysis, combined pretreatment LCs and BMI, age, T/N stage, primary tumor site, alcohol consumption, the presence of comorbidities, and treatment duration were identified as independent risk factors for OS (Table 3).

**Table 2:** Univariate analysis of survival outcomes

Variable	LRFS HR (95.0% CI)	*P*‐value	RRFS HR (95.0% CI)	*P*‐value	DMFS HR (95.0% CI)	*P*‐value	OS HR (95.0% CI)	*P‐*value
BMI/LC		0.157		0.120		0.008^a^		<0.001^a^
(<25.0/≥1950 vs. <25.0/<1950)	0.972 (0.680–1.391)	0.879	0.865 (0.559–1.340)	0.517	0.729 (0.499–1.064)	0.101	0.840 (0.656–1.075)	0.166
(≥25.0/<1950 vs. <25.0/<1950)	0.646 (0.399–1.047)	0.076	0.793 (0.465–1.351)	0.394	0.592 (0.363–0.966)	0.036	0.612 (0.442–0.848)	0.003^a^
(≥25.0/≥1950 vs. <25.0/<1950)	0.715 (0.475–1.078)	0.109	0.514 (0.299–0.886)	0.017^a^	0.481 (0.304–0.761)	0.002^a^	0.485 (0.357–0.658)	<0.001^a^
2010 AJCC T stage (T3–4 vs. T1–2)	1.860 (1.364–2.535)	<0.001^a^	1.315 (0.913–1.893)	0.141	1.825 (1.316–2.532)	<0.001^a^	2.411 (1.924–3.021)	<0.001^a^
2010 AJCC N stage (N2–3 vs. N0–1)	1.443 (1.068–1.949)	0.017^a^	2.228 (1.506–3.295)	<0.001^a^	2.421 (1.714–3.419)	<0.001	1.728 (1.394–2.142)	<0.001^a^
Primary tumor site		<0.001^a^		0.001^a^		0.009^a^		<0.001^a^
(Oropharynx vs. NPC)	1.861 (1.287–2.692)	0.001^a^	1.355 (0.849–2.161)	0.203	1.263 (0.857–1.862)	0.239	2.121 (1.625–2.768)	<0.001^a^
(Hypopharynx vs. NPC)	2.430 (1.669–3.537)	<0.001^a^	2.217 (1.428–3.442)	<0.001^a^	1.574 (1.049–2.362)	0.028^a^	3.489 (2.698–4.513)	<0.001^a^
(Larynx vs. NPC)	1.383 (0.769–2.487)	0.278	0.533 (0.194–1.468)	0.224	0.266 (0.084–0.841)	0.024^a^	1.983 (1.358–2.897)	<0.001^a^
Sex (Male vs. Female)	1.291 (0.857–1.944)	0.222	1.910 (1.073–3.398)	0.028^a^	2.884 (1.601–5.196)	<0.001^a^	2.132 (1.517–2.997)	<0.001^a^
Cigarette smoking (Yes vs. No)	1.791 (1.267–2.533)	0.001^a^	1.889 (1.229–2.902)	0.004^a^	1.732 (1.203–2.495)	0.003^a^	2.111 (1.642–2.713)	<0.001^a^
Betel quid chewing (Yes vs. No)	1.471 (1.094–1.978)	0.011^a^	1.489 (1.037–2.138)	0.031^a^	1.713 (1.255–2.339)	0.001^a^	1.922 (1.564–2.362)	<0.001^a^
Alcohol consumption (Yes vs. No)	1.743 (1.294–2.348)	<0.001^a^	1.693 (1.177–2.435)	0.005^a^	1.587 (1.160–2.172)	0.004^a^	1.889 (1.531–2.330)	<0.001^a^
Comorbidities (Yes vs. No)	1.166 (0.866–1.570)	0.312	1.157 (0.804–1.665)	0.433	0.959 (0.696–1.322)	0.799	1.339 (1.088–1.647)	0.006^a^
Chemotherapy (Yes vs. No)	1.484 (1.012–2.175)	0.043^a^	1.458 (0.910–2.336)	0.117	1.349 (0.892–2.041)	0.157	1.369 (1.039–1.802)	0.025^a^
PET (Yes vs. No)	0.711 (0.504–1.002)	0.052	0.895 (0.572–1.400)	0.627	0.897 (0.611–1.316)	0.578	0.701 (0.553–0.888)	0.003^a^
Age, years^b^	1.007 (0.994–1.020)	0.317	1.010 (0.994–1.026)	0.203	1.005 (0.991–1.019)	0.488	1.032 (1.023–1.041)	<0.001^a^
Treatment duration, days^b^	1.012 (0.978–1.046)	0.506	1.002 (0.960–1.045)	0.939	1.027 (0.992–1.063)	0.136	1.038 (1.015–1.062)	0.001^a^
EQD2, Gy^b^	1.082 (1.000–1.171)	0.050	1.049 (0.951–1.158)	0.342	1.012 (0.928–1.105)	0.785	1.043 (0.986–1.103)	0.144
BMI, kg/m^2^ (≥25.0 vs. <25.0)	0.696 (0.508–0.954)	0.024^a^	0.669 (0.454–0.987)	0.043^a^	0.603 (0.428–0.849)	0.004^a^	0.579 (0.462–0.727)	<0.001^a^
LC, cells/μL (≥1950 vs. <1950)	0.970 (0.723–1.302)	0.840	0.756 (0.526–1.085)	0.129	0.713 (0.521–0.976)	0.035	0.776 (0.632–0.955)	0.016^a^

AJCC, American Joint Committee on Cancer; BMI, body mass index; CI, confidence interval; DMFS, distant metastasis‐free survival; EQD2, equivalent dose in 2 Gy fractions; HR, hazard ratio; LC, lymphocyte count; LRFS, local recurrence‐free survival; NPC, nasopharyngeal cancer; OS, overall survival; PET, positron emission tomography; RRFS, regional recurrence‐free survival.

a *P *<* *0.05.

b Continuous variable.

**Table 3:** Multivariate analysis of survival outcomes

Variable	LRFS HR (95.0% CI)	*P*‐value	RRFS HR (95.0% CI)	*P*‐value	DMFS HR (95.0% CI)	*P*‐value	OS HR (95.0% CI)	*P*‐value
BMI/LC	–	–	–	–	–	0.009^a^	–	<0.001^a^
(<25/≥1950 vs. <25/<1950)	–	–	–	–	0.675 (0.460–0.992)	0.045^a^	0.790 (0.613–1.018)	0.068
(≥25/<1950 vs. <25/<1950)	–	–	–	–	0.587 (0.358–0.963)	0.035^a^	0.610 (0.438–0.850)	0.004^a^
(≥25/≥1950 vs. <25/<1950)	–	–	–	–	0.490 (0.307–0.780)	0.003^a^	0.504 (0.368–0.690)	<0.001^a^
2010 AJCC T stage (T3–4 vs. T1–2)	1.552 (1.122–2.147)	0.008^a^	–	–	1.502 (1.071–2.105)	0.018^a^	1.877 (1.487–2.369)	<0.001^a^
2010 AJCC N stage (N2–3 vs. N0–1)	1.232 (0.903–1.681)	0.188	1.971 (13.26–2.931)	0.001^a^	1.931 (1.357–2.747)	<0.001^a^	1.490 (1.191–1.866)	<0.001
Primary tumor site	–	0.025^a^	–	0.052	–	0.094	–	0.001^a^
(Oropharynx vs. NPC)	1.595 (1.072–2.372)	0.021^a^	1.154 (0.708–1.880)	0.565	0.902 (0.597–1.361)	0.623	1.437 (1.071–1.928)	0.016^a^
(Hypopharynx vs. NPC)	1.811 (1.187–2.764)	0.006^a^	1.665 (1.034–2.679)	0.036^a^	0.890 (0.571–1.389)	0.608	1.886 (1.394–2.551)	<0.001^a^
(Larynx vs. NPC)	1.153 (0.620–2.147)	0.653	0.471 (0.167–1.326)	0.154	0.224 (0.070–0.719)	0.012^a^	1.223 (0.806–1.857)	0.345
Cigarette smoking (Yes vs. No)	1.244 (0.813–1.902)	0.314	1.421 (0.828–2.438)	0.203	1.124 (0.706–1.790)	0.622	1.109 (0.801–1.534)	0.534
Betel quid chewing (Yes vs. No)	0.897 (0.631–1.277)	0.547	0.889 (0.578–1.367)	0.592	1.223 (0.831–1.799)	0.307	1.237 (0.962–1.590)	0.098
Alcohol consumption (Yes vs. No)	1.443 (1.011–2.059)	0.043^a^	1.328 (0.872–2.023)	0.186	1.260 (0.875–1.814)	0.215	1.288 (1.005–1.650)	0.045^a^
Sex (Male vs. Female)	‐	‐	1.300 (0.684–2.469)	0.424	2.317 (1.215–4.421)	0.011^a^	1.253 (0.857–1.832)	0.244
Comorbidities (Yes vs. No)	–	–	–	–	–	–	1.341 (1.076–1.671)	0.009^a^
Chemotherapy (Yes vs. No)	1.312 (0.884–1.949)	0.177	–	–	–	–	1.028 (0.749–1.410)	0.864
PET (Yes vs. No)	–	–	–	–	–	–	0.865 (0.660–1.134)	0.293
Age, years^b^	–	–	–	–	–	–	1.024 (1.013–1.034)	<0.001^a^
Treatment duration, days^b^	–	–	–	–	–	–	1.035 (1.011–1.060)	0.004^a^
EQD2, Gy^b^	1.082 (0.993–1.179)	0.070	–	–	–	–	–	–

AJCC, American Joint Committee on Cancer; BMI, body mass index; CI, confidence interval; DMFS, distant metastasis‐free survival; EQD2, equivalent dose in 2 Gy fractions; HR, hazard ratio; LC, lymphocyte count; LRFS, local recurrence‐free survival; NPC, nasopharyngeal cancer; OS, overall survival; PET, positron emission tomography; RRFS, regional recurrence‐free survival.

a *P *<* *0.05.

b Continuous variable.

### Local, regional, and distant metastasis

Of the 912 patients enrolled in this study, 178 (19.5%) developed local recurrence, 119 (13.0%) developed regional recurrence, and 159 (17.4%) developed DM. The 5‐year LRFS rate for all patients was 79.0%. The 5‐year LRFS rates for Group 1, Group 2, Group 3, and Group 4 patients were 75.7%, 76.1%, 83.2%, and 83.9%, respectively (*P *>* *0.05; Table 2). In the univariate analysis, a pretreatment BMI alone was a significant predictor of LRFS. Other factors, including T/N stage, primary tumor site, cigarette smoking, betel quid chewing, alcohol consumption, and CCRT, were also significant predictors of LRFS (Table 2). In the multivariate analysis, T stage, primary tumor site, and alcohol consumption were identified as independent risk factors for LRFS (Table 3).

The 5‐year RRFS rate for all patients was 85.4%. The 5‐year RRFS rates for Group 1, Group 2, Group 3, and Group 4 patients were 83.0%, 84.0%, 85.4%, and 90.2%, respectively (*P *>* *0.05; Table 2). In the univariate analysis, a pretreatment BMI alone was a significant predictor of RRFS. Other factors, including sex, N stage, primary tumor site, cigarette smoking, betel quid chewing, and alcohol consumption, were also significant predictors of RRFS (Table 2). In the multivariate analysis, only N stage was identified as an independent risk factor for RRFS (Table 3).

The 5‐year DMFS rate of all patients was 81.5%. The 5‐year DMFS rates for Group 1, Group 2, Group 3, and Group 4 patients were 75.7%, 80.6%, 84.4%, and 88.0%, respectively (*P *<* *0.01; Fig. 1 and Table 2). In the univariate analysis, pretreatment LCs and a pretreatment BMI, either alone or in combination, were significant predictors of DMFS (Fig. 1 and Table 2). Other factors, including sex, T/N stage, primary tumor site, cigarette smoking, betel quid chewing, and alcohol consumption, were also significant predictors of DMFS (Table 2). In the multivariate analysis, combined pretreatment LCs and BMI, sex, T/N stage, and primary tumor site were identified as independent risk factors for DMFS (Table 3).

## Discussion

To our knowledge, this is the only study to investigate the prognostic significance of combined pretreatment LCs and BMI in a large number of patients with HNC treated with RT alone or CCRT. Our findings demonstrate the significance and convenience of combined pretreatment LCs and BMI for predicting the prognosis of patients with HNC who are undergoing RT with curative intent. Patients with low pretreatment LCs and a low pretreatment BMI were associated with a poor OS and DMFS. In the multivariate analysis, after adjusting for T/N stage and primary tumor site, combined pretreatment LCs and BMI remained an independent risk factor for OS and DMFS.

Previous studies 10, 11, 12, 13, 16 have demonstrated that LCs can predict the survival outcomes of patients with HNC. Huang et al. 11 analyzed 510 human papillomavirus‐related oropharyngeal cancer patients by utilizing pretreatment LCs with a cutoff value of 1700 cells/μL and reported that high pretreatment LCs were associated with a better progression‐free survival. Rachidi et al. 12, 13 and Valero et al. 12, 13 also reported that patients in the highest tertile of pretreatment LCs had a better OS and disease‐specific survival than those in the lowest tertile. In addition to pretreatment LCs, post‐treatment LCs and LCs during treatment are also potential prognostic factors for survival. Campian et al. 10 evaluated 22 human papillomavirus‐negative patients with HNC and reported that a LC of <500 cells/mm^3^ 2 months after the commencement of CCRT was associated with an earlier disease progression. Cho et al. 16 investigated 70 patients with nasopharyngeal cancer and demonstrated that patients in the lower minimum LC group (cutoff value, 245 cells/μL) were associated with a poorer disease‐specific survival and progression‐free survival.

The precise mechanisms of an association between low pretreatment LCs and a poor prognosis are unknown. However, one possible explanation may be that a low LC is associated with an immunosuppressed state that results in the loss of an antitumor‐specific immune response, such as T‐lymphocyte tumor infiltration 17 or cytotoxic T‐lymphocyte‐mediated antitumor activity 18. This may be a reasonable mechanism by which low pretreatment LCs negatively influenced the OS and DMFS of patients with HNC in our study.

Regarding the effects of pretreatment BMI on the prognosis of HNC, several groups have investigated the association between pretreatment nutritional status and the survival outcome of patients with HNC. Pai et al. 5 reported that a high BMI positively correlated with survival. Takenaka et al. 19 reported that BMI was a prognostic factor for survival, independent of the primary tumor site and tumor stage. Park et al. 19 revealed that overweight patients with a BMI of ≥25.0 kg/m^2^ had a lower HR for death (HR: 0.54) than underweight patients with a BMI of <25.0 kg/m^2^. The above findings were consistent with our study, which revealed a significant survival benefit in patients who were overweight prior to treatment.

In our study, patients were stratified according to pretreatment LCs and BMI, which could be easily performed in daily practice. Significant differences in OS and DMFS were observed between patients with high pretreatment LCs/BMI and those with low pretreatment LCs/BMI. Overweight patients with a BMI of ≥25.0 kg/m^2^ and high pretreatment LCs exhibited lower HRs for death (HR: 0.50) and DM (HR: 0.49) than underweight patients with a BMI of <25.0 kg/m^2^ and low pretreatment LCs. Although in the present cohort we demonstrate that patients with low pretreatment LCs and a low BMI are associated with a more advanced T stage, and patients with more aggressive disease and greater tumor burden may have a compromised nutritional status, which limits treatment efficacy, combined pretreatment LCs and BMI remains an independent risk factor for predicting OS and DMFS, as shown in the multivariate analysis. Our findings could facilitate further research of introducing neoadjuvant chemotherapy or early nutritional and hematopoietic colony‐stimulating factor intervention in patients with low pretreatment LCs and a low pretreatment BMI in order to improve the poor OS and DMFS rates relative to patients with high pretreatment LCs and a high pretreatment BMI.

This was also the only study to present nearly 80.0% of the study population as having undergone PET for tumor staging, which makes our findings associated with DMFS more reliable, as the uncertainty in DM status was considerably reduced 20. There are concerns that low pretreatment LCs and a low pretreatment BMI may be related to inefficient CCRT. In pretreatment hematological testing for the feasibility of chemotherapy treatment in our facility, a medical oncologist monitors the white blood cell counts, neutrophil counts, hemoglobin levels, and platelet counts. Although LCs and BMI were considered factors associated with the immune response and nutritional status, they were not incorporated into our pretreatment checklist. Consequently, low pretreatment LCs and a low pretreatment BMI may not have influenced the decision to treat with CCRT. The implementation rate of CCRT, as shown in Table 1, revealed no significant differences between the four arms, which could support our statement.

A major strength of our study was the inclusion of a large number of patients with adequate follow‐up durations and comprehensive staging workups, which included almost 80.0% of the study population having undergone PET for the initial staging.

However, despite performing a careful review of the data, due to its retrospective nature, this study is subject to several inherent limitations, including the fact that we did not present data on the history of immunosuppression or chronic steroid use, and we did not thoroughly check the HPV infection status for patients with head and neck cancer, so the actual prevalence of HPV in our cohort is uncertain. Moreover, the existence of unmeasured confounders, such as subjective treatment decisions, could potentially have introduced bias into the analysis.

In conclusion, our data demonstrate that combined pretreatment LCs and BMI may be more effective at predicting OS and DMFS in patients with HNC treated with RT. Future, large‐scale, prospective clinical trials are warranted to validate our findings.

## Conflict of Interests

None declared.
